# Spinal cord injury: pathophysiology, possible treatments and the role of the gut microbiota

**DOI:** 10.3389/fmicb.2024.1490855

**Published:** 2024-12-18

**Authors:** Luis H. Pagan-Rivera, Samuel E. Ocasio-Rivera, Filipa Godoy-Vitorino, Jorge D. Miranda

**Affiliations:** ^1^Physiology Department, University of Puerto Rico Medical Sciences Campus, San Juan, Puerto Rico; ^2^Microbiology and Medical Zoology Department, University of Puerto Rico Medical Sciences Campus, San Juan, Puerto Rico

**Keywords:** spinal cord trauma, dysbiosis, gut microbiome, gut-brain axis, neurodegenerative

## Abstract

Spinal cord injury (SCI) is a devastating pathological state causing motor, sensory, and autonomic dysfunction. To date, SCI remains without viable treatment for its patients. After the injury, molecular events centered at the lesion epicenter create a non-permissive environment for cell survival and regeneration. This newly hostile setting is characterized by necrosis, inflammation, demyelination, axotomy, apoptosis, and gliosis, among other events that limit locomotor recovery. This review provides an overview of the pathophysiology of SCI, highlighting the potential role of the gut microbiota in modulating the inflammatory response and influencing neurological recovery following trauma to the spinal cord. Emphasis on the bidirectional communication between the gut and central nervous system, known as the gut-brain axis is given. After trauma, the gut-brain/spinal cord axis promotes the production of pro-inflammatory metabolites that provide a non-permissive environment for cell survival and locomotor recovery. Therefore, any possible pharmacological treatment, including antibiotics and painkillers, must consider their effects on microbiome dysbiosis to promote cell survival, regeneration, and behavioral improvement. Overall, this review provides valuable insights into the pathophysiology of SCI and the evolving understanding of the role of the gut microbiota in SCI, with implications for future research and clinical practice.

## 1 Introduction

Spinal Cord Injury (SCI) is a devastating condition caused by damage to the spinal cord or the spinal nerve roots within the spinal canal, which can result in temporary or permanent loss of movement and somatosensory sensation. The leading causes of SCI are vehicle accidents, followed by tragic falls, sports injuries, and diseases that can damage the spinal cord, such as tumors and spinal stenosis (National Spinal Cord Traumatic Statistical Center, 2023). The severity of this neurological state is highly variable in its etiology, for instance, contusion versus transection, the depth of the contusion, and the duration of compression. Also, its level of injury, which is under the anatomical location of the lesion, determines the degree of movement deprivation. For example, severe damage received in the cervical region of the spinal cord will impair movement and function in all extremities, and this state is known as quadriplegia (Maynard et al., 1997), versus lesions induced from the mid-thoracic vertebrae region or lower results in paraplegia (Chay and Kirshblum, 2020). Moreover, SCI could affect the function of several other systems, like the gastrointestinal tract, resulting in intestinal dysfunction, complicating the patient’s well-being and exacerbating the detrimental events that take place at the lesion epicenter through inflammatory cytokines released from the gut (Sweis and Biller, 2017; Jing et al., 2021a).

In the last decade, an increase in reported SCI cases has been noted, affecting roughly 54 people per one million in the United States, and it is estimated that between two to three million people worldwide live with a post-SCI-related disability (Quadri et al., 2020). The incidence is almost four times higher in males than females, with a higher prevalence in adults. Additionally, it is reported that patients with SCI die prematurely in comparison with the rest of the population. This is more evident in countries with mean lower incomes (National Spinal Cord Traumatic Statistical Center, 2023). Spinal cord injuries also present a high economic burden, ranging from several hundred thousand dollars to one million dollars in the first year after SCI and averaging ninety thousand dollars each subsequent year (National Spinal Cord Traumatic Statistical Center, 2023). To date, SCI remains without a cure, and available treatments are focused mainly on improving the patient’s quality of life (Fan et al., 2018). In this review, we highlight recent findings on the pathophysiology of SCI from pre-clinical and clinical trials, how changes in gut microbiome composition affect cell survival, locomotor recovery, drug absorption, and how the possibility of microbiota-targeted therapeutic strategies combined with drugs that show positive outcomes after SCI can be a viable novel therapeutic technique for SCI.

## 2 SCI pathophysiology

**Figure 1:**
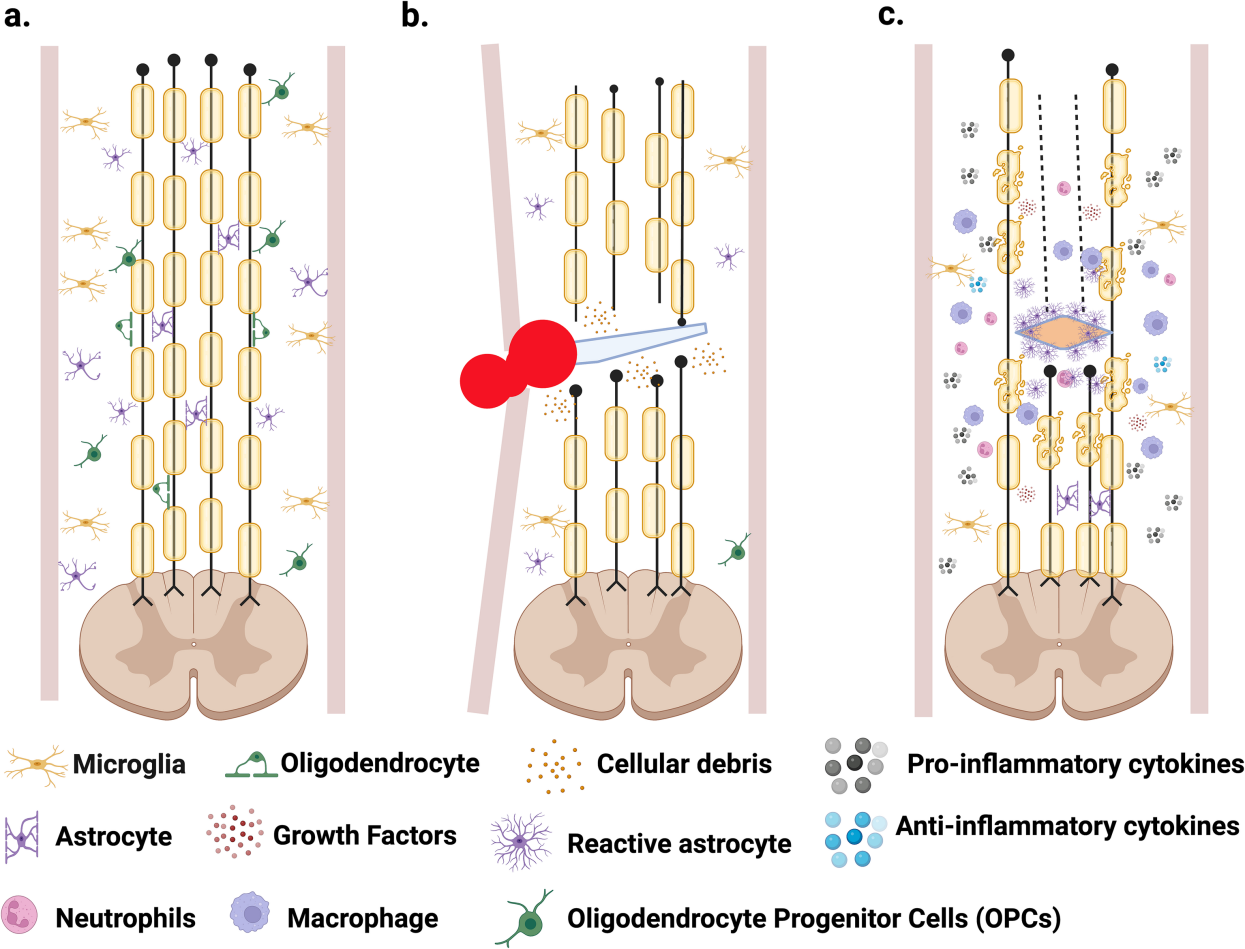
Spinal cord injury progression. **(A)** Healthy spinal cord consisting of multiple neuronal cells. **(B)** Primary phase caused by trauma to the spinal cord, resulting in necrosis, axotomy, hemorrhage, edema, and immune cells infiltration that promotes inflammation. **(C)** Secondary phase is characterized by astrogliosis and the formation of the glial scar, demyelination and apoptosis, with the continuous inflammatory response. Created in BioRender. Pagan (2024) https://BioRender.com/r98w142.

The complex nature of SCI results in a detrimental and non-permissive environment at the lesion epicenter that inhibits regeneration and cell survival. Several molecular and cellular events occur at the trauma site, which are the major factors for the poor progression in finding a viable cure for SCI. Normally, spinal cord physiology involves interactions between many neuronal cells, such as neurons, astrocytes, microglia, oligodendrocyte progenitor cells (OPCs) and oligodendrocytes (Figure 1A). After SCI, these interactions are altered and interrupted, resulting in clinical symptoms and arduous recovery (Couillard-Despres et al., 2017). Spinal cord injury is divided into two phases: primary and secondary injury. The primary phase is due to the sudden trauma in the spinal cord, characterized by events such as hemorrhage, edema, and axotomy (Figure 1B). The physical insult may produce cell death of neurons, astrocytes, oligodendrocytes, microglia, and endothelial cells through necrosis.

The rupture of blood vessels results in the extravasation of red and white blood cells, which increases pressure at the lesion site, further disrupting the blood flow (Alizadeh et al., 2019) and contributing to ischemia (Alizadeh et al., 2019). This affects local neurons and glial cells, which are deprived of oxygen and nutrients, resulting in cellular death (Quadri et al., 2020). The absence of oxygen reduces the production of ATP, affecting the Na^+^/K^+^ pump and increasing sodium ions inside the cells, promoting water influx and cell swelling. These cellular events may also promote cell death (Quadri et al., 2020). In addition, depending on the severity of the trauma, axotomy may occur. The high number of inhibitory proteins expressed after SCI and the low/appropriate trophic support create a non-permissive environment for cell survival and regeneration (Van Niekerk et al., 2016). Cellular events triggered during the primary phase of SCI are difficult to solve since they are initiated by the physical impact and the timing makes it impossible to treat.

In the secondary phase, further damage is observed with events such as apoptosis, demyelination, inflammation, and astrogliosis that form a glial scar (Hellenbrand et al., 2021) (Figure 1C). This phase starts hours after the injury and may extend for several months, leading to more cellular death and damage, and a non-functional spinal cord (Hellenbrand et al., 2021; Domingues et al., 2016). Understanding its complex pathophysiology is essential for developing a viable cure for SCI (O’Shea et al., 2017). Some of the main events that take place during this secondary phase are discussed briefly below:

1. Glutamate is a key excitatory neurotransmitter of the central nervous system (CNS) and causes excitotoxicity if released uncontrollably. Its receptors, N-methyl-D-aspartate (NMDA), *α*-amino-3-hydroxy-5-methyl-4-isoxazolepropionic acid (AMPA), and kainite, are expressed throughout the spinal cord and are involved in motor activity and nociceptive pathways (Bardoni, 2013). After SCI, excitotoxicity due to uncontrollably glutamate released, and hyperactivation of NMDA and AMPA receptors will lead to increased Ca^++^ and Na^+^ ions entry, which promotes cell death (Blesch and Tuszynski, 2009; Park et al., 2004). Targeting these receptors has become a key focus in neuroprotection and recovery research. In a 2024 study, Yan et al. modeled excitotoxic injury by exposing primary spinal cord neurons from neonatal rats to high concentrations of glutamic acid. Transcriptomic analysis revealed six significantly upregulated genes *in vitro*, which were also elevated in rats with subacute SCI. Notably, two genes, Hspb1 and Lgals3, were closely associated with excitotoxicity-induced autophagy. These findings provide new insights into the interplay between excitotoxicity and autophagy, suggesting novel targets for diagnosing and treating SCI (Yan et al., 2024).
2. Apoptosis is evident the following hours to weeks after the lesion in neurons, glia, and oligodendrocytes (Beattie et al., 2000). In essence, apoptosis is programmed cellular death, a regulated response after a stimulus arising from the surrounding cellular environment, internal metabolism, or even the cellular genome (Lou et al., 1998). Studies have shown that apoptosis contributes to tissue damage after SCI and results in the loss of important neural cells, increasing the damage caused by the initial injury (Hellenbrand et al., 2021). Therefore, mechanisms that inhibit or modulate apoptosis after SCI may provide clinical implications in the development of viable treatment (Shi et al., 2021), as demonstrated by Xu et al. (2019) when a reduction in the pro-apoptotic gene Bax, locomotor recovery was promoted after SCI.
3. Demyelination describes the loss of myelin, which occurs after damage to the CNS. Myelin functions as an insulating layer that forms around the nerves. It prevents the loss of Na^+^ ions that travel along the nerve axon during the conduction of an action potential and is crucial in maintaining the appropriate speed of the nerve impulse that proceeds towards the synaptic bulb to trigger neurotransmitter release and provides its distinct saltatory conduction (Salzer, 2015). Spinal cord injury decreases the velocity in the conduction of the messages that travel along the axon because of a deterioration of the myelin sheath and cell death of oligodendrocytes (Wang et al., 2012). Trauma to the spinal cord causes necrosis and apoptosis of oligodendrocytes due to the physical contact or glutamate excitotoxicity manifested in the secondary phase, as mentioned above (Anjum et al., 2020). Oligodendrocyte loss leads to demyelination of the axons, inhibiting axonal function by blocking the saltatory action potentials (Domingues et al., 2016). Concurrently, proteins that are expressed in the myelin are well-known inhibitors of axonal outgrowth (Löw et al., 2008). Among those proteins are NOGO, MAG, and OMgp, which have been characterized as blockers of axonal regeneration after SCI (McKerracher and Rosen, 2015).
4. Astrocytes are specialized glial cells and are the most abundant in the CNS (Sofroniew and Vinters, 2010). These specialized cells have many important roles in CNS physiology. For instance, they connect to blood vessels through projections on their end-feet, and in this way, they contribute significantly to the formation and maintenance of the Blood Brain Barrier (BBB) and Blood Spinal Cord Barrier (BSB) (Donati et al., 2005). Astrocytes also protect from neurotoxicity and cell death by up-taking GABA, glycine, and glutamate neurotransmitters from the synaptic cleft (Bundesen et al., 2003). Lastly, astrocytes also assist in neurotransmitter synthesis and neural metabolism, and collectively, they possess distinctive cellular properties that are integral to the normal functioning of the CNS (Sofroniew and Vinters, 2010). After SCI, astrocytes experience molecular, cellular, and functional changes (Figure 1C) that include an increase in Glial Fibrillary Acidic Protein (GFAP) and vimentin expression, especially in astrocytes located near the injury site (Eng et al., 2000). This process in which astrocytes undergo reactive changes in response to injury is known as astrogliosis, and it is considered another of the pathogenic hallmarks of SCI (Karimi-Abdolrezaee and Billakanti, 2012), because the glial scar forms a physical and chemical barrier for axonal regeneration (Cregg et al., 2014). The degree of the astrocytic gliosis reaction relies on SCI severity, time, and astrocyte location relative to the lesion site (Brambilla et al., 2005). Like the inflammatory response, astrogliosis presents some benefits after the lesion, and multiple studies have shown astrocytes possess a critical protective role after SCI (Sofroniew and Vinters, 2010; Sofroniew, 2009; Cho, 2009). Reactive astrocytes initiate a response to limit the flow of peripheral leukocytes to the lesion site, reduce the lesion cavity’s expansion, and help activate microglia to assist the damaged BSB (Sofroniew, 2005). They also aid with glutamate uptake, as mentioned above, limiting excitotoxicity. Through the secretion of antioxidants, like glutathione, astrocytes also limit oxidative stress after SCI (Bush et al., 1999).

Studies performed by Faulkner et al. (2004) demonstrated that inhibition of reactive gliosis in animal models affected blood-spinal cord barrier reconstruction after SCI, proving how astrocyte activation is essential in minimizing the damage after SCI. On the other hand, reactive astrocytes contribute to the non-permissive, hostile environment generated in the lesion area after SCI through the secretion of inhibitory factors (Egnaczyk et al., 2003) that affect axonal growth (Goldshmit et al., 2011). Reactive astrocytes produce proteoglycans of the extracellular matrix (ECM) and express proteins such as Eph, ephrins, and semaphorins that, together, create a dense glial scar at the lesion area that presents a physical and chemical barrier for axonal regeneration (Profyris et al., 2004). Astroglial scarring is one of the limiting factors for axonal regeneration after SCI (Okada et al., 2018). Reactive astrocytes in the glial scar produce chondroitin and keratin sulfate proteoglycans, which are inhibitory molecules that limit the axonal regeneration after SCI, and treatment with chondroitinase has resulted in the degradation of chondroitin, allowing axonal outgrowth (Silver and Miller, 2004).

5.Some beneficial effects of inflammation are known after SCI. For example, removing pathogenic microbes that can cause infection and promoting the healing process after the lesion is crucial. However, damage after inflammation stems from the extensive and prolonged infiltration of immune cells, leading to tissue impairment and promoting further destruction (Glaser et al., 2004). This important process is achieved through multiple cell types and inflammatory cytokines such as Interleukin-1β (IL-1β), Interleukin-6, and Tumor Necrosis Factor (TNF). These cytokines and other chemokines are released by microglial cells, astrocytes, and immune cells that infiltrate the lesion site (García et al., 2016). As mentioned, this prolonged infiltration of immune cells, such as neutrophils and macrophages, contributes toward neural degeneration. Furthermore, their presence at the lesion site contributes to the production of additional inflammatory mediators such as cytokines, prostaglandins, and glycoproteins (Garcia et al., 2016). The severity of the primary lesion greatly contributes to the degree of inflammation, which can often result in an inflammatory overreaction (Figure 1C), causing additional cellular death (DiSabato et al., 2016).

In summary, SCI is a multifactorial event characterized by molecular and cellular changes that take place at the lesion epicenter, as well as in the lesion penumbra, producing a non-permissive environment for cell survival and a repulsive milieu for axonal regeneration. In addition, the pathology is complicated because the changes that affect the spinal cord start immediately after the contusion and continue months after the initial insult. Therefore, the changes are dynamic and progressive. Moreover, trauma to the spinal cord not only disturbs the communication within the CNS but also affects other systems like the cardiovascular (Nash and Gater, 2020), respiratory (Josefson et al., 2021), renal (Romo et al., 2018) and gastrointestinal system (Bernardi et al., 2020).

Among the problems expressed by many SCI patients is the suffering of neurogenic bowel dysfunction (NBD), and approximately 11% of patients who suffer SCI return to the hospital because of problems with the gastrointestinal tract (Jaglal et al., 2009). The main reason for NBD is the lack of neural control over the gastrointestinal system, and the two major results are incontinence or lack of control to release feces and constipation. The reduction in the intestinal motility after SCI could result in a change in the gut microbiota. Gungor et al., 2016 studied the gut microbiota of SCI patients with different types of NBD and observed that total bacterial counts of some genera were significantly lower in NBD groups versus healthy individuals (Gungor et al., 2016). The intestinal flora imbalance was also investigated by Lin et al. (2020), when this group analyzed the feces of SCI and healthy patients and observed that the structure and quantity of gut microbiota were different among the studied groups. Several studies confirmed that the gut microbiome contributes to the normal function of living vertebrates through the metabolisms of the ingested food, maintenance of the bacterial flora diversity, proper development of the immune response system, suitable intestinal epithelial cells barrier, and nutrient absorption, which could be feasible to speculate that SCI results in a gut dysbiosis that aggravates the patient lifestyle. Thus, the disruption of gut microbiome diversity (dysbiosis) may alter the balance of prokaryotes in the intestinal tract, affecting metabolite production (Kong et al., 2023) or appropriate immune response, resulting in a reduction in the functional locomotor recovery after SCI through different mechanisms (Kigerl et al., 2016). Moreover, the authors (Kigerl et al., 2016) observed that the detrimental effects caused by using a broad-spectrum antibiotic exacerbate the pathophysiology after SCI, which this could be reverted with commercial probiotics. Therefore, a multi-active drug is necessary to target many of the molecular and cellular events initiated after SCI to promote cell survival, axonal outgrowth, and locomotor recovery, among other beneficial results. However, any effect of a potential drug to treat SCI patients should consider its effect on the gut microbiome (Kigerl et al., 2016).

## 3 SCI and gut microbiome

Studies have shown that the chronic use of pharmacological compounds has adverse implications for the gut microbiome, a collection of trillions of microbes that reside in the human digestive tract, focused mainly on the large intestine (Reynoso-García et al., 2022). In its homeostatic state, the gut microbiome has several effects on human health, such as promoting innate and adaptive immunity (Dethlefsen et al., 2007), maintaining intestinal epithelial integrity (Kim et al., 2012), and aiding in metabolism in synthesizing essential nutrients such as vitamins and carbohydrates (Morowitz et al., 2011). The normal otherwise “healthy” gut microbiome is the most diverse of the human body niches (Reynoso-García et al., 2022). It is characterized by a wide variety of microbial species, including a high diversity of bacteria and other microorganisms, such as unicellular fungi and viruses, that provide a key role in resisting pathogens (Yin et al., 2019). Changes in the natural “healthy” state of the microbiome are known as dysbiosis, an imbalance that could be due to the gain or loss of community members and functions due to changes in the relative abundance of microbes (Bidell et al., 2022).

Prolonged dysbiosis leads to gut inflammation and a dysregulated immune system, and an overall reduction in gut alpha diversity estimates. Dysbiosis has been associated with the loss of protective *Bacteroides, Bifidobacterium* and *Faecalibacterium* and a rise in taxa within the Proteobacteria phylum—now Pseudomonadota, which can increase the risk of systemic infection, including bacteremia (Lawley and Walker, 2013). Intestinal inflammation may be further exacerbated with dysbiosis, with a decreased production of short-chain fatty acids (SCFAs), such as butyrate, that provide anti-inflammatory effects and help maintain gut barrier integrity (Kho and Lal, 2018). Pang et al. (2022) analyzed the gut microbiome of 23 spinal cord injury (SCI) patients and 21 healthy individuals. Their goal was to explore the connection between gut microbiome changes and lymphocyte subsets. They discovered that SCI patients had a significantly higher gut microbiota diversity index compared to healthy controls. However, butyrate-producing bacteria like Fusobacterium, which benefit gut health, were markedly reduced in SCI patients. Correlation analysis indicated that five bacterial genera in SCI patients were linked to T lymphocyte subsets and NK cells. These findings suggest that gut microbiota in SCI patients is closely related to lymphocyte subsets, indicating that modulating the gut microbiome could help correct immune dysregulation and potentially offer a novel therapeutic approach for SCI (Pang et al., 2022).

**Figure 2:**
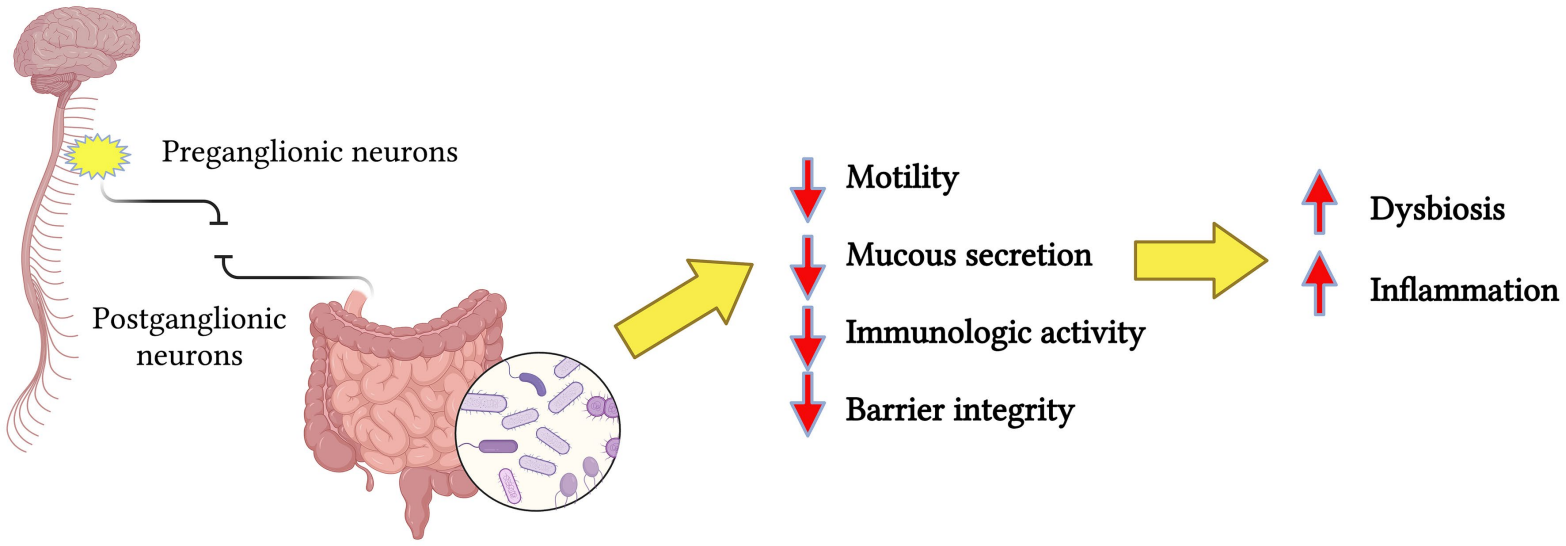
Simplified representation of spinal cord injury, resulting in the disruption of preganglionic and postganglionic neurons communication, causing the loss of neural input over the gastrointestinal tract. The end-result is a change in the microbiota profiles resulting in dysbiosis and inflammation. Created in BioRender. Pagan (2024) https://BioRender.com/a13d580.

Growing evidence suggests that the gut microbiota has a crucial role in the bidirectional communication between the gut and the central nervous system (Cui et al., 2024). This interaction occurs via various pathways including the vagus nerve, immune system mediators, microbial metabolites, and neurotransmitters that can cross the blood–brain barrier. Results indicate that gut microbes may shape neural development and modulate neurotransmission, likely contributing to the pathogenesis of many neurological conditions (Socała et al., 2021). For example, evidence indicates that gut dysbiosis may advance the pathogenesis of Alzheimer’s disease, promoting amyloid-beta aggregation, neuroinflammation, oxidative stress, and insulin resistance (Brandscheid et al., 2017). This bidirectional signaling within the gut-brain axis also involved microbially derived short-chain fatty acids (SCFAs), such as butyrate, propionate, and acetate, which are metabolic byproducts of microbial fermentation processes in the gut. These SCFAs have been shown to exert anti-inflammatory effects and to protect against neurodegeneration (Castillo-Álvarez and Marzo-Sola, 2022). Jing et al. (2023a) demonstrated that patients with SCI had reduced levels of specific SCFAs. Supplementing these SCFAs reduced inflammation and promoted the repair of neurological tissues, resulting in improved functional recovery. Likewise, the imbalance of the microbiota-gut-brain axis has been studied in Parkinson’s disease (PD). The overstimulated immune system reaction due to gut dysbiosis, accompanied by enhanced intestinal permeability, provokes systemic inflammation. At the same time, the activation of enteric glial and neuronal cells may contribute to the production of *α*-synuclein (α-syn) aggregates, a hallmark in PD (Dogra et al., 2022). Similarly, traumatic brain injury (TBI) promotes gut dysbiosis and intestinal barrier dysfunction that contributes to the development of systemic inflammation and the secondary phase of central nervous system injury (Chiu and Anderton, 2023). Additionally, gut dysbiosis aggravates behavioral impairment in both TBI and SCI animal models, as well as murine stroke models (Rice et al., 2019). SCI could affect the sympathetic preganglionic neurons in the thoracic spinal cord, disrupting the control and homeostasis of the postganglionic neurons that innervate the gastrointestinal tract (Kigerl et al., 2018). Loss of this neural input over the GI will lead to the impairment of motility, mucous secretion, immunologic activity, and the integrity of the epithelial barrier, causing bacterial translocation resulting in gut dysbiosis (Gungor et al., 2016) (Figure 2).

Bazzocchi et al. (2021) examined and characterized the gut microbiota of 100 SCI patients within a short time, no later than 60 days after SCI. To undergo this analysis, feces were collected within the first week at the rehabilitation center and profiled by 16S rRNA gene-based next-generation sequencing. The microbial profile results were analyzed and compared to those publicly available of healthy age and gender-matched populations. The gut microbiota of SCI patients showed signs of dysbiosis, such as an increase in potentially pathogenic, pro-inflammatory bacteria such as *Streptococcaceae* and a decrease in short-chain fatty acid producer taxa including *Ruminococcaceae*. Another significant finding was how the dysbiosis varies by lesion and severity degree, with the most neurologically impaired patients showing an even more unbalanced microbial profile. The observation of the increased gut permeability and inflammation may potentially predispose patients to the onset of more severe complications associated with SCI, such as microbial translocation, immunosuppression, urinary tract irregularities, and neurogenic bowel dysfunction (NBD) (Ahuja et al., 2017).

Neurogenic bowel dysfunction is a common SCI complication with symptoms that include constipation and fecal incontinence (Magnuson et al., 2023). Zhang C. et al. (2018) focused on determining the association of gut microbiome in NBD after SCI. A small cohort of 43 SCI patients, 20 quadriplegics, and 23 paraplegics, along with 23 healthy male adults, was used. Stool samples were collected from all the participants, and interviews were conducted to survey the NBD management. Gut microbiota profile analysis was completed by sequencing the V3-V4 region of the 16S rRNA gene. Results found that patients with quadriplegia took a longer time to defecate in comparison with paraplegia and healthy subjects. Regarding microbial gut diversity, both SCI groups (quadriplegic and paraplegic) showed decreased diversity and reduced bacterial structural composition. Specifically, the SCI groups showed an increase in both Veillonellaceae and Prevotellaceae bacterial families. These changes in bacteria composition are associated with the inflammatory processes and the presence of lipopolysaccharides (LPS), respectively (Forbes et al., 2016; Larsen, 2017). Additionally, Bacteroidaceae family and *Bacteroides* genera decreased in the SCI group. Metatranscriptomic analysis have shown how the Bacteroidaceae bacterial family is involved in many bacterial functional genes in the active gut microbiota, such as the catabolism of carbohydrates, highlighting its crucial role of microbiome homeostasis (Rosas-Plaza et al., 2022). At the same time, *Bacteroides* help to provide protection from pathogens (colonization resistance) and supply nutrients to other microbial residents of the gut (Zafar and Saier, 2021). The study of Zhang C. et al. (2018) and Zhang J. et al. (2018), highlighted the microbial community structure, the resulting dysbiosis, and its association with a prolonged defecation time in SCI patients who suffer from NBD. Another study by Yu et al. (2021) confirmed the results mentioned previously. The reduction in the diversity of the gut microbiota correlated with the NBD score of patients with complete SCI versus patients with incomplete SCI and composition distinct from healthy individuals, suggesting that the gut diversity could be related to the degree of SCI (Yu et al., 2021).

Dysbiosis after SCI can be manifested by a decrease in the abundance of the Bacillota phylum (former Firmicutes), with or without the increase of Bacteroides phylum, resulting in the change of the ratio of Firmicutes to Bacteroides (Grigoreva, 2021). This change in microbiota structure reduces the diversity of protective taxa, which is followed by a potential pathogenic or pro-inflammatory taxa increase, affecting the production of short-chain fatty acids (SCFAs) that are one of the main bioactive mediators of the gut microbiota (Xiao et al., 2022). These bacterial changes lead to gut dysfunction and an increased inflammatory response, affecting patient recovery (Liu et al., 2023). Liu et al. (2023) studied the effect of the oral administration of exogenous SCFAs in Sprague–Dawley rats after SCI. A mixture of acetate, propionate, and butyrate was added to the water for 21 days. Rats given the SCFAs mixture increased the BBB open-field locomotor score (which measures locomotor recovery). ELISA, qPCR, and immunohistochemistry analysis showed reduced spinal cord tissue inflammation, and the spinal cord necrosis cavity was reduced. An increase in Interleukin-10 (IL-10) expression was observed in the spinal cord of treated rats with SCFAs, which plays a role in maintaining gut homeostasis (anti-inflammatory cytokine). On the other hand, a decrease in Interleukin-17 (IL-17), a pro-inflammatory cytokine, was noticed in the spinal cord. In addition, this study shows how SCFAs promoted gut homeostasis but also how SCFAs induced intestinal T cells to shift toward an anti-inflammatory phenotype. This study did not provide a microbiome composition analysis. Still, the reported findings suggest a relationship between the gut, spinal cord, immune cells, and the production of SCFAs by providing mechanisms for regulating neural repair after SCI. Bannerman et al. (2021) expanded these findings to explain the possible contribution of microbiome dysbiosis and neuroinflammation after SCI to the development of pain in patients with spinal cord trauma (Bannerman et al., 2021). In a study by Jing et al. in 2021, they induced SCI in mice to study how fecal matter transplants (FMT) can exert a neuroprotective effect after SCI. Fecal matter transplant is a method to directly change the recipient’s gut microbiota to normalize the composition and gain a therapeutic benefit, reverting dysbiosis (Wang et al., 2019). BBB open field test scores, immunohistochemistry analysis, and microbiome profile comparison showed that the mice who received FMT presented functional recovery and improved neuronal axonal regeneration (Khoruts et al., 2010). Moreover, high-throughput sequencing analysis revealed that levels of phylum Bacillota were reduced in fecal samples of SCI mice, and FMT helped to reshape the gut microbiome. Lastly, FMT-treated SCI mice showed increased fecal short-chain fatty acids (SCFAs). This study showed how managing dysbiosis can be a promising route for SCI treatment.

Evidence shows that gut microbiota pro-inflammatory metabolites can enter the central nervous system via the blood-spinal cord barrier, resulting in neuroinflammation and contributing to the secondary phase of SCI. Rong et al. (2021) conducted to analyze the correlation between gut microbiota and inflammatory processes after SCI. Trauma to the spinal cord was exerted in mice by contusion after performing a T10 laminectomy, and fecal matter was collected weekly. The 16S amplicon sequencing was used to identify the diversity and abundance of gut microbes. ELISA was used to detect the serum levels of pro-inflammatory and anti-inflammatory factors. Western blots and qRT-PCR were used to investigate the expression of the TLR4/MyD88 signaling pathway. Results showed differences between SCI and healthy mice, where the gut of SCI mice presented dysbiosis, accompanied by increased levels of pro-inflammatory cytokines such as necrosis factor-*α*, interleukin (IL)-1*β*, and IL-6, coinciding with an activation of the TLR4/MyD88 signaling pathway. Also, the expression of anti-inflammatory factors IL-4, transforming growth factor-β, and IL-10 were decreased (Rong et al., 2021). This study showed how gut dysbiosis caused by SCI can activate the TLR4/MyD88 signaling pathway, resulting in an increased inflammatory response after SCI. Kang et al. (2023) integrated metabolomics analysis to investigate the correlation between gut microbiota and metabolites, along with the possible mechanisms underlying the effects of gut microbiota on secondary injury after SCI. In this study, SCI was induced with T8–T10 contusion in mice. Then, 16S rRNA gene amplicon sequencing from fecal samples and metabolomics analysis using Liquid Chromatography-Electrospray Ionization Tandem Mass Spectrometry (LC-ESI-MS/MS) from spinal cords was conducted to reveal the changes and/or correlation in gut microbiota with metabolites in the spinal cord. Results showed a severe gut microbiota dysbiosis after SCI, evidenced by an increase in pro-inflammatory bacteria genera such as *Shigella*, *Rikenella*, *Staphylococcus*, and *Mucispirillum* and decreases in anti-inflammatory bacteria, such as *Lactobacillus*, Allobaculum, and *Sutterella* (Table 1).

**Table 1:** Levels of bacteria populations in normal murine and after SCI organized as pro-inflammatory or anti-inflammatory according to the results presented in different research papers.

	Bacteria genera	Control	SCI	Published article
Pro-inflammatory bacteria	Shigella	↓	↑	Kang et al. (2023)
	Rikenella	↓	↑	Kang et al. (2023)
	Staphylococcus	↓	↑	Kang et al. (2023)
	Anaerotruncus	↓	↑	Valido et al. (2022)
	Butyricimonas	↓	↑	Jing et al. (2021b)
	Weissella	↓	↑	Du et al. (2021)
	Lactococcus	↓	↑	Du et al. (2021)
Anti-inflammatory bacteria	Allobaculum	↑	↓	Kang et al. (2023)
	Sutterella	↑	↓	Kang et al. (2023)
	Faecalibacterium	↑	↓	Valido et al. (2022), Zang et al. (2023)
	Megamonas	↑	↓	Valido et al. (2022)
	Roseburia	↑	↓	Valido et al. (2022)
	Streptococcus	↑	↓	O’Connor et al. (2018)
	Clostridium	↑	↓	O’Connor et al. (2018)
	Ruminococcus	↑	↓	O’Connor et al. (2018)
	Faecalibacterium	↑	↓	O’Connor et al. (2018)
	Turicibacter	↑	↓	Du et al. (2021)

Note that some taxa classified as pro-inflammatory can often be found in healthy animals/humans.

A review of research manuscripts revealed some conflicting bacterial population changes as well as classification criteria such as anti-inflammatory or pro-inflammatory taxa, as this always depends on the whole microbiota at the time of the experiment; dysbiosis is often a community phenotypic response. For example, O’Connor et al. (2018) showed a decrease in *Clostridium* genus after SCI, coupled with an increase in pro-inflammatory interleukin IL-1β. Contrarily, Jing et al. (2019) reported an increase in *Clostridium* after SCI. Many factors could contribute to this discrepancy; for instance, O’Connor et al. (2018) showed microbiome data analysis 8 weeks after SCI, whereas Jing et al. (2019) conducted analysis 4 weeks after SCI. Another notable difference was antibiotic administration, while O’Connor et al. (2018) administered the antibiotic gentamicin through 1 week after the lesion, Jing et al. (2019) in the meantime induced microbiome dysbiosis through an antibiotic cocktail of ampicillin, neomycin, and metronidazole 2 weeks before SCI. Additionally, both groups followed the murine animal model, albeit O’Connor group worked with female rats, and Jing used female mice. Experimental design differences impact reported microbiome composition after SCI and should be accounted for in assigning pro- and anti-inflammatory roles in the bacterial genera. Lastly, except for *Shigella,* all others can also be anti-inflammatory depending on which other bacteria surround them (Al Bander et al., 2020; Ndungo et al., 2022).

Gut microbiota studies after SCI suggest that any treatment to improve behavioral recovery must also reduce dysbiosis caused by the lesion and/or drug treatment. The administration of probiotics after peripheral traumatic nerve crush injury in mice proved to revert the dysbiosis caused by the administration of antibiotics, increasing *Akkermansia* genera related to the production of anti-inflammatory SCFAs (Rodenhouse et al., 2022). This study shapes the possibility of administering pro-biotics for SCI treatment after dispensing antibiotics following nerve damage lesions.

### 3.1 Change in microbiome can affect drug absorption

**Figure 3:**
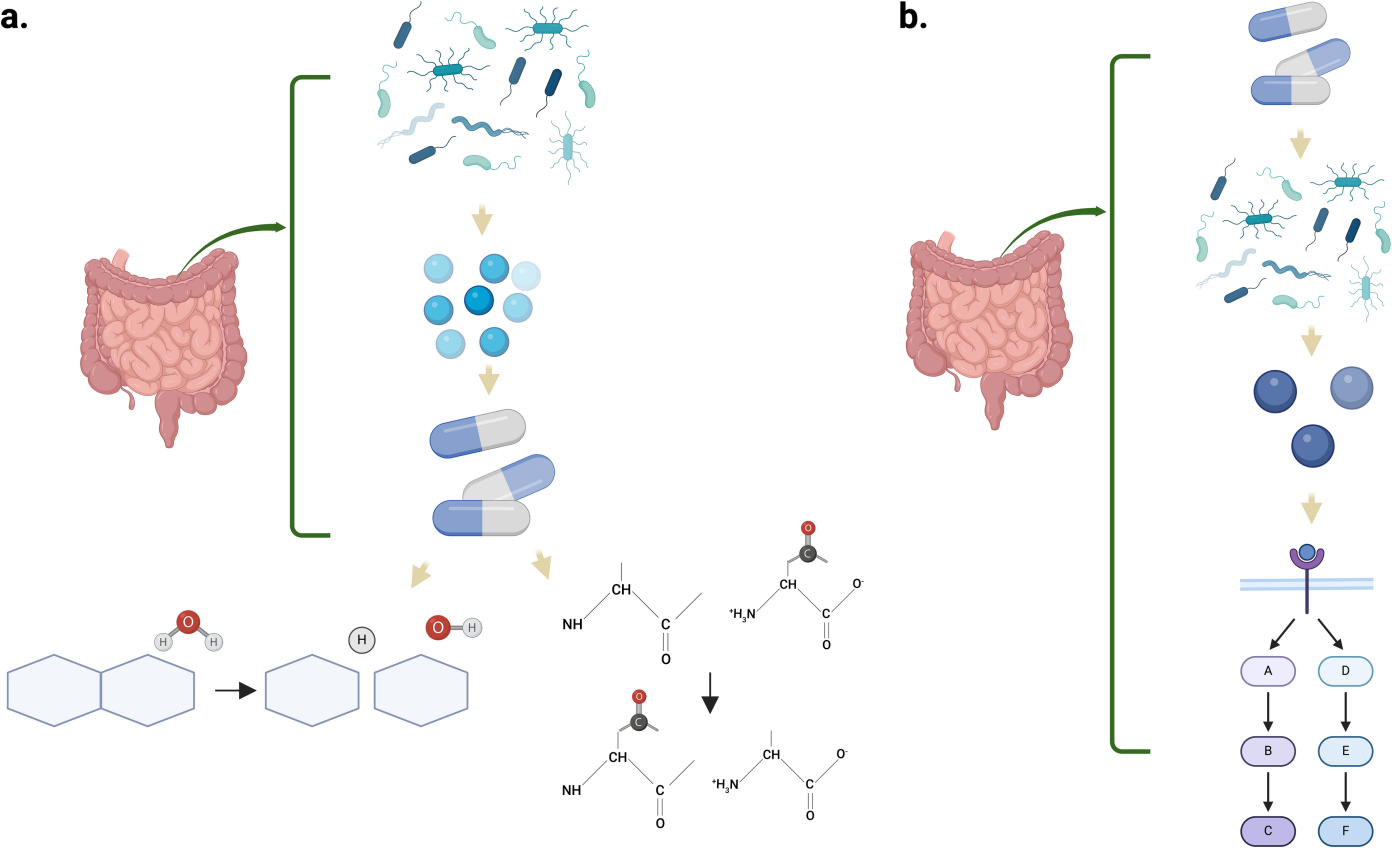
Mechanisms by which the gut microbiome can metabolize internalized drugs. **(a)** Enzymes synthesized by the microbes metabolize the drugs through multiple biochemical reactions such as hydrolysis and functional group transfer. **(b)** Microbial metabolites interact with host receptors, triggering complex signaling pathways that influence drug response and therapeutic outcomes. Created in BioRender. Pagan, L. (2024) https://BioRender.com/z34y623.

The microorganisms that compose the gut microbiome contribute with the modification of drugs administered orally or systemically (Tsunoda et al., 2021). Among the drug transformations encountered by the microbiome are activation, inactivation/degradation, stability, or toxicity, and these transformations could result in altered treatment outcomes (Pant et al., 2023). Two general mechanisms have been identified by which the gut microbiome metabolizes the internalized drugs. One mechanism is using the microbial enzymes that directly metabolize the drug. Among the metabolic reactions mediated by the gut microbiome enzymes are reduction, hydrolysis, functional group transfer, and cleavage (Figure 3A) (Liu et al., 2024). The second is an indirect pathway through metabolites produced by the microorganisms in the gastrointestinal tract that interacts with the host receptors, activating specific signaling pathways (Figure 3B). Recently, there has been an increase in the research community to understand the role of the microbiome in the pharmacokinetics of drugs used for the treatment of several conditions and the therapeutic outcomes (Zhang et al., 2021). As discussed by Zhang C. et al. (2018) and Zhang J. et al. (2018), drugs like amiodarone (antiarrhythmic) and levodopa are metabolized by the gastrointestinal microbiome, enhancing their therapeutic efficacy (Zhang J. et al., 2018). On the other hand, enzymes from the altered intestinal microflora could also inactivate drugs and/or generate toxic metabolites that are not beneficial for the intestinal mucosal barrier (Li et al., 2020). Subramaniam et al. (2023) demonstrated that self-emulsifying drug delivery systems (SEDDS) disrupt Sprague Dawley rats’ gut microbiome after a 21-day administration of a representative pharmaceutical formulation. This disruption was characterized by a reduction in alpha diversity, including observed operational taxonomic units (OTUs) as measured by Shannon’s index, and a statistically significant shift in beta diversity distances. The resulting dysbiosis increased pro-inflammatory cytokine levels, contributing to intestinal barrier injury and potentially impairing future drug absorption (Subramaniam et al., 2023).

During dysbiosis, there is an alteration in the gut microflora that promotes induction of harmful bacteria, for instance, members of the Proteobacteria/Pseudomonadota phylum, which affect the absorption of drugs or its active metabolites and reduce the activity of specific transporters in the gastrointestinal epithelia. SCI can exacerbate dysbiosis by disrupting gut motility and immune regulation, leading to an imbalance in microbial composition. This imbalance can impair drug absorption and metabolism, potentially reducing therapeutic efficacy or causing adverse effects (Cui et al., 2024). However, treatment with antibiotics or non-antibiotic drugs could also induce dysbiosis and may induce the acquisition of antimicrobial resistance, affecting the pharmacokinetics of therapeutical drugs (Maier et al., 2018). Moreover, the gut microbiome could affect the bioaccumulation of therapeutical drugs inside the bacterial cells and should also be considered because it reduces the availability of the drug.

SCI-related dysbiosis can further compromise neuro-recovery by promoting systemic inflammation, which negatively influences spinal cord healing. Additionally, disruptions in the gut-brain axis due to SCI can impair neurotransmitter regulation, further complicating drug responses and overall recovery outcomes (Loh et al., 2024). Thus, individual microbiome analysis must be considered before the administration of any drug to improve the response of a medication and the life quality of the patient (Mousa et al., 2023). Therefore, possible drugs that reduce inflammation, demyelination, gliosis, or compounds that interact with specific channels to reduce excitotoxicity should consider any effect on the microbiome, including co-treatment with antibiotics. This point is relevant for the treatment of SCI, since at least 24% of drugs that are not antibiotics produce changes in the gut microbiome and may interfere with the final outcome that is expected (Maier et al., 2018).

## 4 Possible pharmacological treatments

Spinal cord injury treatment consists mainly of surgery with antibiotics and medications for pain control (Liu et al., 2021). Laminectomy has been proven to be the most effective surgery intervention for SCI patients, helping to relieve compression in the lesion area (Rouanet et al., 2017). Other procedures, such as open decompression of the spinal cord and spinal dura mater, are also frequently used for clinical treatment. However, one of the main reasons SCI remains a complicated scenario for its patients is that many cells at the lesion site and its penumbra could follow an apoptotic pathway and/or are exposed to pro-inflammatory cytokines (Fehlings and Vawda, 2011). This states the grounds for pharmacological intervention as another route of treatment, taking advantage sometimes of the hydrophobicity of a drug or the temporary rupture of the blood-spinal cord-barrier after SCI. Methylprednisolone (MP) has been shown to inhibit the production of the TNF-*α*, IL-Iβ, and IL-Iα inflammatory factors in the secondary phase of SCI (Costa et al., 2015). Studies have shown that the administration of MP from 8 to 24 h after SCI leads to improved neurological function (Sultan et al., 2020). Nevertheless, treatment with this drug remains controversial along with their therapeutic dosage because high doses of MP can lead to adverse reactions in patients, such as blurred vision, gastric bleeding, and menstrual changes, and for those reasons, it is not used anymore (Liu et al., 2019). The drugs that must be used clinically to treat SCI patients should primarily improve the environment in the injured area, protecting neurons and slowing nerve cell detriment (Costa et al., 2015).

The administration of neurotrophic factors after SCI in the research environment has made great progress in recent years because these factors can reduce neuronal apoptosis, promote neuronal regeneration, and induce synapse formation (Harvey et al., 2015). The blood–brain barrier has been shown to affect the concentration of neurotrophic factors that can reach the injured area, and high-dose administration is required (Wilson et al., 2013). Reis et al. (2018) used fibroblast growth factor 2 (FGF-2) encapsulated into core-shell microfibers to treat SCI in rat models. Results showed that its administration supported the survival and proliferation of PC12 cells *in vitro* and increased the locomotor recovery of the rats 4 weeks after SCI, indicating a possible route of administration for these factors. BDNF and NT-3 have also shown significant improvement in terms of sprouting, axonal elongation, and neuroprotection after SCI (Fang et al., 2017; Keefe et al., 2017).

Several neuroprotective and neurodegenerative agents targeting pathological mechanisms are currently undergoing clinical trials. Riluzole is a sodium-glutamate antagonist that attenuates neurodegeneration in amyotrophic lateral sclerosis (ALS), and it has also been shown to promote neurological functional restoration after SCI (Fehlings et al., 2023; Fehlings and Vawda, 2011). In pre-clinical models of SCI, Riluzole reduced the secondary phase by blocking the activation of sodium channels and reducing the release of neuronal glutamate (Schwartz and Fehlings, 2002). Early-phase clinical trials have shown favorable results in promoting recovery in these pre-clinical models of SCI (Fehlings et al., 2023). Additional animal studies support the effect of steroidal hormones like estradiol (Mosquera et al., 2014), testosterone (McLoughlin et al., 2023), and progesterone (Jure et al., 2019) as potential neuroprotective agents after SCI. Moreover, the use of selective estrogen receptor modulators (SERMs) after SCI has been demonstrated to increase spared tissue, reduce the glial scar, and produce functional locomotor recovery (Colón et al., 2016; Colón et al., 2018).

Pharmacotherapy against molecules that block axonal outgrowth, or regeneration has also been studied. Agents, like chondroitinase ABC, that reduce the amount of chondroitin sulfate proteoglycans in the glial scar promote corticospinal tract sprouting and behavioral recovery in animal models with SCI (Yousefifard et al., 2022). Moreover, blocking NOGO or semaphoring-3A reduces the repulsive environment at the lesion epicenter, promoting axonal regeneration, sprouting, and functional recovery (Wang et al., 2015; Yoshida et al., 2024). Cethin is a permeable paste that is applied to the dura of the spinal cord after SCI, and it carries the BA-210 bacterial toxin that inhibits the Rho GTPase pathway of inhibitory proteins. Studies have shown that the Rho GTPase pathway is activated after SCI (Dubreuil et al., 2003). Bao et al. (2004) also observed how the Rho GTPase pathway regulates cell migration and the production of inflammatory cytokines such as tumor necrosis factor-α (TNF-α), interleukin-1 beta (IL-1β), interleukin-2 (IL-2). A phase I/IIa trial was undertaken by administrating Cethrin to the dura membrane during surgery in 48 patients with complete SCI. No complications were documented against the drug at 1-year follow-up, and the patients receiving 1 and 3 mg doses showed improvement (Fehlings et al., 2011). As of 2021, another subset of patients with acute traumatic cervical SCI are being enrolled in the United States and Canada to test the efficiency of VX-210, formerly known as BA-210 or Cethrin. Neurological and functional changes will be evaluated at 6 weeks and at 3, 6, and 12 months after (Fehlings et al., 2021). Additional studies in animal models towards serotonergic-related therapy (Heijmans et al., 2021) and purinergic receptors (Cheng et al., 2023) also present promising results after SCI. However, the effect of these possible pharmacological agents on the microbiome is unknown, and the role of drug therapy in dysbiosis has not been investigated and should be established for the benefit of SCI patients.

To avoid any dysregulation of the intestinal microbiota or to balance the gut microbiome, compounds that re-establish the intestinal flora should be considered after SCI (Willman et al., 2022). The use of probiotics or melatonin has been considered and studied with beneficial outcomes (Zhang et al., 2022; Kigerl et al., 2016). In addition, possible interventions with the transplantation of fecal matter to re-establish a healthy microbiota should be considered, since pre-clinical studies in animal models demonstrated a reduction in gut dysbiosis after TBI and SCI (Du et al., 2021; Jing et al., 2023b).

### 4.1 Therapeutic approaches

The blood–brain barrier (BBB) is a sophisticated and highly selective boundary that plays a crucial role within the neurovascular unit of the central nervous system (CNS). This specialized structure shields neurons from direct exposure to circulating neurotoxic substances, pathogens, and peripheral inflammatory processes mainly due to the presence of tight junction proteins such as claudins, occludins and tricellullin (Abbott et al., 2010). A decreased expression of theses tight junction proteins, accompanied by an increased BBB permeabilization have been addresses through the comparison of germ-free mice with normal gut microbiome (Braniste et al., 2014). The gut microbiota generates various molecules and metabolites that can influence the host’s central nervous system (CNS) either positively or negatively. Short-chain fatty acids (SCFAs), such as propionate, butyrate, and acetate, are produced through the bacterial fermentation of non-digestible polysaccharides in the lower intestine, these SCFAs function as signaling molecules, possess anti-inflammatory effects, and help protect colonic epithelial cells (Kigerl et al., 2018). Maintenance of non-toxic baseline levels of short-chain fatty acids (SCFAs) can help preserve the integrity of the intestinal barrier, protect the blood–brain barrier (BBB) from oxidative damage, and promote the expression of tight junction proteins (Fock and Parnova, 2023).

As discussed, SCI leads to a decrease in SCFAs-producing bacteria. This reduction has also been observed in other CNS pathologies such as hepatic encephalopathy (Shahbazi et al., 2023). SCFAs produced by gut microbiota also influence the central nervous system (CNS) through the enteric nervous system (ENS), which typically communicates with the CNS via the vagus nerve and sympathetic pathways (Needham et al., 2020). Since gut dysbiosis might play a role in the pathophysiology BBB disruption in SCI, therapeutic approaches targeting gut dysbiosis and its metabolites could help restore the damaged BBB. This, in turn, may reduce immune cell infiltration, oxidative stress, and inflammation associated with SCI. Antibiotic therapy targets the gut microbiota, and accumulating evidence indicates that a number of antibiotics such as macrolides, minocycline and beta-lactams exert anti-inflammatory and neuroprotective effects in different CNS pathologies, including SCI (Afshari et al., 2021). Schmidt et al. (2021) studied the effect of minocycline on the gut microbiota and systemic immune response after spinal cord injury. Results showed that minocycline was found to significantly and rapidly impact gut microbiota diversity and composition, coinciding with the normalization of cytokine and chemokine levels suppressed by SCI. Notably, SCI-induced gut dysbiosis has been associated with anxiety-like behavior, which was also reduced by minocycline treatment, lastly, while minocycline mitigated microglial activation caused by SCI, they did not observed a reduction in lesion size or lead to noticeable improvements in motor recovery (Schmidt et al., 2021). Still, the impact of antibiotics on maintaining BBB integrity is quite limited. Thabut et al. (2015) showed that administering rifaximin to hyperammonemic bile duct ligation rats significantly reduced the fluorescent intensity of fluorochrome in brain tissue, indicating decreased BBB permeability. However, it’s become clear that the management and modulation of the gut microbiome using some therapeutic options, such as probiotics and prebiotics (Kigerl et al., 2018; Toh et al., 2020), antibiotic therapy (Toh et al., 2020) and fecal microbiota transplantation (Jing et al., 2021b) have beneficial effects on the neuroprotection, maintaining tissue integrity and locomotion recovery after SCI. Future exploration of these validated therapies, combined with strategies to maintain BBB integrity, may offer a broad spectrum of effective management options for SCI.

## 5 Conclusion

Spinal cord injury results in a physical, emotional, psychological, and financial impact on patients and their families. At the molecular level, SCI creates a hostile environment that hinders cell survival and regeneration, preventing effective recovery of locomotor function. It also alters the diversity of the gut microbiota, and this dysbiosis exacerbates the non-permissive environment at the injury site due to its pro-inflammatory effects. Lastly, the resulting dysbiosis impacts the metabolism of drugs that could benefit patients, highlighting the importance of incorporating probiotics as a key component of treatment protocols. There is limited data on the therapeutic potential of any pharmacological drug in maintaining a healthy microbiome after SCI. Pre-clinical studies coupling SCI with microbiome and immune responses will certainly help develop novel therapeutic approaches, and more studies are needed. Moreover, possible drug treatments to reduce the detrimental effects produced after SCI and to promote functional locomotor recovery must consider its effect on the patient microbiome.
